# The state of postpartum contraceptive use in India: descriptive lessons from nationally representative survey data

**DOI:** 10.1186/s12978-025-01978-3

**Published:** 2025-03-13

**Authors:** Nicole E. Johns, Abhishek Singh, Shruti Ambast, Nandita Bhan, Katherine Hay, Vedavati Patwardhan, Lotus McDougal

**Affiliations:** 1 Center On Gender Equity and Health, School of Medicine, University of California San Diego, 9500 Gilman Drive, La Jolla, CA USA; 2 Department of Public Health and Mortality Studies, International Institute for Population Sciences, Mumbai, Maharashtra India; 3 Jindal School of Public Health and Human Development, O.P. Jindal Global University, Sonipat, Haryana India

**Keywords:** Postpartum contraception, Postpartum family planning, India, Contraceptive use, Postpartum period

## Abstract

**Background:**

Postpartum contraception is a key tool to delay or prevent subsequent pregnancy after birth. Though prior research has demonstrated substantial dynamism in contraceptive use throughout the postpartum period, most measurement of postpartum contraception has focused on aggregate use of any method at a single time point. We sought to more thoroughly examine the continuum of postpartum contraceptive use amongst women in India.

**Methods:**

We use 2019–21 National Family and Health Survey reproductive calendar data from n = 149,518 women with a birth in the one to five years prior to survey. We present estimates of postpartum contraceptive use by month postpartum, use of specific methods, initiation, duration, stopping, method switching, and subsequent pregnancy. We examine sociodemographic and birth factors associated with postpartum contraceptive use using multivariate logistic regression models. We also examine patterns of postpartum utilization for subpopulations of interest (adolescent mothers age 15–19 and first time mothers) and test whether conclusions are sensitive to a two-year rather than one-year postpartum time period definition.

**Results:**

We find that 59% of Indian women used a method of contraception within the first year postpartum, that condoms and female sterilization were the most commonly used methods, and that patterns of postpartum contraceptive use differed substantially by month, method, and subpopulation. Among postpartum contraceptive users, 9% switched methods, 19% stopped using contraception entirely, and 5% had another pregnancy within the first year postpartum. A number of sociodemographic and birth factors are associated with postpartum contraceptive utilization, and patterns of use differ meaningfully for adolescent and first-time mothers. Most findings were consistent when using a two-year rather than one-year time frame.

**Conclusions:**

The dynamic nature of postpartum contraceptive use suggests limited value of static contraceptive uptake targets, whether for program planning or as measures of success, and bolsters the need to center and to improve reproductive agency, empowerment, and access throughout the postpartum period.

**Supplementary Information:**

The online version contains supplementary material available at 10.1186/s12978-025-01978-3.

## Plain English summary

After birth (postpartum), many women want to prevent or delay getting pregnant again. Postpartum contraception can help enable women to do so. Most research on postpartum contraception just looks at whether women used anything to prevent or delay a pregnancy at any point within the one to two years after birth, but we know that women often start, stop, and change methods in that time frame. We used nationally-representative data from India collected in 2019–21 to more closely examine the ways in which women use contraception after birth. We found that 3 in 5 women used a method within the first year, most commonly condoms or female sterilization, but that the patterns of use (such as when they started using a method, which method(s) they used, and how long they used for) differed by a number of factors. Specifically, we found different patterns of use based on which month after birth women began a method, based on the specific method used, and for specific groups of women (such as adolescents age 15–19 or first-time mothers). Women who did use contraception postpartum frequently switched methods, stopped using contraception, or became pregnant again within a year, suggesting that measuring postpartum contraceptive use as only a yes/no outcome fails to capture how dynamic this time period is. Measuring and setting targets for women to use contraception after birth should include more nuanced questions that capture this change in order to focus on and improve women’s reproductive agency, empowerment, and contraceptive access during the postpartum window.

## Background

Contraception is recognized as key enabler for improving the health and wellbeing of women and children worldwide [1–3]. Unintended and closely-spaced pregnancies are associated with a range of negative maternal and child health outcomes, including maternal and infant mortality, delayed or reduced used of antenatal care, prenatal and postpartum depression, maternal experience of interpersonal violence, preterm birth, low birth weight, and stunting, among others [4–10]. In addition to these health impacts, contraception supports individuals to achieve their desired family size and to space pregnancies at preferred intervals, enabling agency over whether, with whom, when, and how often to have children. Though contraception is important to consider at all stages of the reproductive life cycle, it is particularly relevant for women who have recently given birth. The use of contraception after birth can prevent a closely-spaced subsequent pregnancy and the resultant negative health impacts of a short inter-pregnancy interval for women who would like to delay or avoid a subsequent pregnancy. Prior research suggests that while most women want to wait at least one year before becoming pregnant after a birth, many do not initiate contraceptive use within that time frame, resulting in substantial rates of unmet need for contraception among postpartum women [11, 12]. Additionally, many women access the health system before, during, and after delivery, creating potential touch points for providers to share information on and access to contraception and related services.

Though the terms ‘family planning’ and ‘contraception’ are often used interchangeably in the literature and in policy, they reflect two distinct constructs [13]. *Contraception* is the use of methods to prevent pregnancy, though contraceptives can be used for reasons other than pregnancy prevention such as to suppress menstruation or relieve symptoms of related disorders such as premenstrual dysphoric disorder. *Family planning* (FP) includes contraception, but also includes procedures, behaviors, and enabling conditions which allow people to obtain their desired number of children and preferred spacing of pregnancies, inclusive of infertility treatments and abortion. Though both concepts are relevant in the postpartum period, we focus here on postpartum contraception (PPC) specifically.

Increasing the use of PPC has been the target of a number of policy initiatives globally. The provision of immediate PPC has been recognized as a high-impact practice [14], and substantial efforts have been made to integrate PPC education and provision into antenatal and postnatal care, childhood immunization services, and other existing maternal and child health service delivery pathways [15–19]. However, there has been mixed evidence of the effectiveness of these interventions; one review suggests that receipt of any prenatal care, home visitation programs during the postpartum period, and educational/counseling interventions are generally associated with improved postpartum contraceptive uptake, but that mother-infant care integration and cash transfer programs warrant further investigation [20]. While uptake of PPC is the primary outcome metric for most evaluated interventions and policies [21], the postpartum user journey (whether—and what method—to use, when to start, whether to switch methods, and whether to stop) is less well understood.

Better understanding of contraceptive use throughout the postpartum period can inform more specific and person-centered interventions to improve contraceptive outcomes, beyond the aim of improving uptake generally. Measurement of PPC is non-standardized across data sources, and generally conceived as a cross-sectional snapshot of contraceptive use at a given number of months postpartum. This approach fails to capture the substantial variation in initiation, method switching, and discontinuation which may occur throughout the postpartum time period. Previous research from Malawi, for example, demonstrated notable change in contraceptive use during the first 12 months postpartum, including nearly 50% discontinuation of the initially used method and subsequent switching to an alternate method [22]. Despite this sizeable volume of change, most analyses of PPC utilize a single metric of use at a specific time point (e.g. at 12 months postpartum) or of use at any point within a given postpartum window. Greater understanding of when women start using a method, how long they use for, what method they switch to, and why they discontinue using is critical to designing more person-centered initiatives to support reproductive agency in the postpartum time frame.

In India, an estimated 6% of all women of reproductive age are postpartum in a given year (translating to more than 22 million women in 2023) [23, 24]. Data from the National Family and Health Survey (NFHS) 2015–16 suggest that 45.6% of postpartum women used a method of contraception within 12 months of birth, a substantially lower rate of use than among women of reproductive age generally, 53.5% [25]. To our knowledge, there are no available estimates of PPC use globally or within the South Asian region specifically, however one review from low and middle income country (LMIC) contexts suggested a comparable *modern* PPC use rate of 41.2% in LMICs, and 42.4% in the South/South East Asian region specifically [21]. The overall use of contraception in India has increased in the most recent 2019–21 wave of NFHS, to 66.7% among all women of reproductive age [26]. Despite this increase in use, more than a quarter of births (27%) occurred within 24 months of the preceding birth (e.g. before the WHO-recommended birth interval) [26]. The large absolute number of postpartum women, lower than average postpartum contraceptive use, and substantial proportion of closely-spaced births suggests a need to understand women’s choices and patterns of use in the Indian context, and ultimately to support reproductive agency and birth outcomes in the postpartum time frame. Historically, contraceptive policies in India have focused on delay of first birth and limiting total family size, with less focus on reversible contraceptive methods or the nuances of dynamic contraceptive use over time [27]. Greater consideration and understanding of the continuum of contraceptive use, inclusive of methods which allow for spacing of subsequent births, is particularly relevant for a postpartum population.

Two populations are of particular programmatic interest for PPC programs in India, namely adolescent mothers and first-time mothers. Broadly, these groups demonstrate consistently lower contraceptive utilization than older and multiparous women [26, 28]. Married adolescents, defined as age 15–19, are particularly vulnerable, as they are more likely to have lower decision-making power within their marriages and households [29–31], lower educational attainment [32], lesser knowledge of or access to FP services [28, 33], and poorer marital and reproductive health outcomes [34]. Despite lower-than-average rates of contraceptive utilization within India, recent work suggests an increase in postpartum contraceptive use among young women from 33% in 2015–16 to 42% in 2019–21, coupled with increases in pregnancy health service utilization [35]. While first-time mothers may also be adolescents, most are not (India’s median age at first birth is 21) [26]. Programmatic interest around first-time mothers primarily centers around the fact that these women are often younger and recently married, with a focus on ensuring desired and safe spacing of the first birth [27, 36–38]. Though little programmatic work specifically targets these women *after* their first birth, this window may be a time at which contraceptive use becomes more normatively acceptable, given fertility expectations early in marriage [39–41]. The transition to parenthood is also time of high stress, during which the healthcare needs of parents may be secondary to ensuring healthcare and childcare for the newborn; reaching first-time parents may therefore require a more proactive approach for PPC services.

The objectives of this work are as follows: (a) to summarize the continuum of PPC use for women in India, inclusive of use by month postpartum, use of specific methods, and initiation, continuation, length of use, stopping use of contraception, switching of methods, and subsequent pregnancy; (b) to summarize the continuum of PPC use for populations of interest including adolescents and first-time parents; and secondarily, (c) to examine sociodemographic factors associated with PPC use; and (d) examine how PPC use outcomes differ when utilizing alternative postpartum time frame definitions.

## Materials and methods

### Data source

Data for this study come from the 5th round of the National Family Health Survey (NFHS-5) conducted in 2019–2021 [42]. NFHS-5 is a nationally-, state-, and district-representative household survey which collects a wide range of information on population, health, nutrition, and related constructs. NFHS-5 utilized a stratified two-stage sampling design, first stratifying each of 707 included districts by urban and rural areas and selecting primary sampling units (PSUs) based on census enumeration blocks (urban) or villages (rural) within each strata. PSUs with 300 or more households were segmented to clusters of 100–150 households each. In the second stage, 22 households per PSU or segment of PSU were randomly selected for interview. NFHS-5 includes four components, namely Household, Woman, Man, and Biomarker Questionnaires. Data for this study were collected as part of the Women’s Questionnaire, obtained from all eligible women age 15–49 in included households. Extensive detail on NFHS-5 survey methodology has been published elsewhere [26].

### Defining the postpartum time period

We examine the postpartum time period using contraceptive calendar (also termed reproductive calendar) data [43]. In the contraceptive calendar, women are asked to recall pregnancies, births, terminations and contraceptive use monthly for at least five years prior to survey. The calendar is completed and verified throughout the Women’s Questionnaire, including prompts to ascertain timing of births and terminations, length of pregnancies, and dates and duration of contraceptive method use. Each month has a value corresponding to a birth, termination, pregnancy, specific type of contraceptive use, or none of the above. Each month in the recall window in which a woman reports discontinuing a method (either to stop using any method, switch to a new method, or because she became pregnant), she is also asked the reason for discontinuation; this information is captured in linked discontinuation calendar data.

Contraceptive methods included in the NFHS-5 contraceptive calendar are: female sterilization, male sterilization, intrauterine device (IUD)/PPIUD, injectables, pill, condom/Nirodh, female condom, diaphragm, foam or jelly, Lactational Amenorrhea (LAM), rhythm method (also known as the calendar method or fertility awareness method, where women abstain from sex on days in which they’re likely to be fertile), withdrawal, “other modern methods”, or “other traditional methods”. Prompts provided to describe each of these methods can be found in Annex Document 1. Due to low rates of use (≤ 0.1% of the study population) and to avoid censoring of the few observations with uncommon method use, we grouped the following methods as ‘Other methods’: male sterilization, female condom, diaphragm, foam or jelly, “other modern methods”, and “other traditional methods”.

The postpartum time frame can be defined in multiple ways. Common intervals include 12 weeks [44], six months [45], and one year after birth [46]; the WHO also recommends pregnancy spacing of at least two years after a live birth before attempting the next pregnancy, with contraceptive implications for that window [47]. We examine PPC using a one year after birth time frame for all primary analyses. This analysis of PPC utilization included all currently married women with a pregnancy between 12 and 59 months prior to survey. We thus include all married women with complete follow-up for the postpartum time period. No further exclusion criteria were applied. When women had multiple eligible births within the postpartum time frame (only 0.3% of the sample), the most recent birth was analyzed.

For each woman included in the study sample, we analyzed the 12 months of calendar data following the reference birth to examine patterns of contraceptive utilization and subsequent pregnancy.

As a sensitivity analysis, we replicated key analyses utilizing a two-year postpartum time frame definition. This sample included all married women with a pregnancy between 24 and 59 months prior to survey, and these sensitivity analyses included the 24 months of calendar data following the reference birth.

### Study population

NFHS-5 included a total of 747,176 eligible women age 15–49, of whom 724,115 provided response to the Women’s Questionnaire (response rate 97%). Of these, 152,143 women (21% of the total women’s sample) had a birth in the 12–59 months prior to survey and 149,518 of these (99% of all women with a birth) were married at time of survey, constituting the 12-month PPC sample. 124,830 women (17%) had a birth in the 24–59 months prior to survey and 122,571 (99% of all women with a birth) were married at time of survey, constituting the 24-month PPC sample for sensitivity analyses. The 12-month sample ultimately included births from July 2014 to April 2020; the 24-month sample included births from July 2014 to May 2019.

### Outcome variables

To understand PPC use, we examine a number of related metrics. Unless otherwise noted, all women are censored upon a subsequent pregnancy (regardless of termination or birth outcome). For example, if a woman becomes pregnant six months postpartum, she is counted as part of the postpartum sample in months one through five, and for any 12-month overall tabulations. She would be excluded from month-specific analyses examining months six or later postpartum, as she would be pregnant, rather than postpartum, at that time. PPC rates therefore represent contraceptive use postpartum and before a subsequent pregnancy.

Any contraceptive use in the postpartum time frame: First, we examine an indicator of whether women reported use of any contraceptive method in any of the 12 months following birth. We also examine any use of each specific contraceptive method during any of the 12 months following birth.

Contraceptive use by month postpartum: We then examine contraceptive use rates overall and by method type for each specific month postpartum.

PPC initiation: We examine average time to initiation of PPC, overall and by specific method. We also report percent of women initiating PPC at each specific month postpartum.

Duration of PPC use: We present the percent of PPC users continuously using their method for at least six months, among those who initiated within the first six months to ensure complete follow-up data availability.

Stopping PPC use: We examine what percent of PPC users subsequently stopped contraceptive use entirely for at least one month within the postpartum time frame, overall and by method.

Switching PPC methods: We report what percent of PPC users switched to using an alternate method within the postpartum time frame, reporting both direct switching (no months of non-use in between methods) as well as switching with one or more months of non-use in between.

Subsequent pregnancy: We report the percent of women who had a subsequent pregnancy within the postpartum time frame.

PPC discontinuation: We define discontinuation as an umbrella term comprising stopping PPC use entirely for at least one month of non-use, switching PPC methods, or subsequent pregnancy. We report reasons for discontinuation overall and by method.

For outcomes which may have occurred multiple times in the postpartum time frame (for example, initiation, discontinuation, and re-initiation of a single method), the first instance was analyzed (for example, the first instance of initiation of the method). Multiple occurrences of such outcomes were rare; the noted example occurred in less than 0.01% of the sample.

### Lactational amenorrhea validation

Lactational amenorrhea (LAM) is a contraceptive method defined for women [1] whose menses have not returned since birth, [2] who are exclusively breastfeeding, and [3] who are within six months postpartum [48]. In the NFHS contraceptive calendar, women are asked to self-report LAM use, though often do not meet the three criteria for LAM to be considered a valid method; prior research suggests that only 26% of self-reported LAM users in similar surveys meet the criteria for correct LAM practice [49]. As part of the Women’s Questionnaire, women are separately asked to report how long they were amenorrhoeic after each birth and how long they breastfed their most recently born child. For their most recent birth, they were also asked whether they gave their infant anything other than breastmilk in the first three days after birth. Though exclusive breastfeeding was not directly ascertained for past births, we used this first three days item as a proxy for exclusive breastfeeding (likely over-estimating true LAM utilization). To have verified LAM use in a contraceptive calendar month, women must have reported amenorrhea for at least as many months after birth (e.g. at least 4 months of amenorrhea if reporting LAM in month 4 postpartum), must have reported breastfeeding for at least as many months after birth (e.g. at least 4 months of breastfeeding if report LAM in month 4 postpartum), must not have given their child anything other than breastmilk in the first three days after birth, and must have been six or fewer months postpartum. Self-reported LAM data which met all three criteria were retained as LAM use, while self-reported LAM data which did not meet at least one criterion were recoded as non-use of any method.

No other modifications to calendar data were made.

### Additional variables of interest

Sociodemographic factors: Sociodemographic factors included age at time of birth (continuous and two-level 15–19 vs 20–49 years), age at first cohabitation (continuous [censored to 10 years if < 10 years reported] and two-level < 18 vs 18 + years), education (none, primary, secondary, or higher), wealth quintile, and residence (urban, rural).

Birth-level factors: Birth-level factors related to the index birth included parity (1, 2, 3, 4 or more), child sex (male, female), pregnancy intention (intended, wanted to wait, or wanted no more children), 4 or more ANC visits (yes or no), and facility delivery (public health facility delivery; private health facility (including NGO health facility) delivery; or home delivery or ‘other’ delivery location). We also examined length of continuous abstinence after birth; women who reported ‘unknown’ or ‘inconsistent’ months for this survey item or who were missing item response (n = 2121, 0.8% of sample) were considered non-abstinent (0 months).

### Analyses

We first summarize rates of PPC use for the postpartum population as a whole, overall, by method, and by month. We also present contraceptive use and method mix for the married women of reproductive age (WRA) population as a whole for comparison.

We then summarize rates of initiation, continuation, stopping, switching, and subsequent pregnancy, for PPC users overall and by method.

For two key populations of interest, adolescent mothers age 15–19 and first-time mothers, we present greater detail on PPC use, including initiation, continuation, switching, and subsequent pregnancy.

We then present bivariate comparisons of PPC use overall by individual sociodemographic and birth-related factors, using adjusted Wald tests (continuous measures) or Pearson’s chi-squared tests (binary/categorical measures) as appropriate. We also present adjusted associations with PPC overall and by method, utilizing multivariable logistic regression models.

We finally present the sensitivity analysis utilizing a 24-month postpartum time window, replicating key analyses as outlined above.

All analyses were conducted using Stata 18.0. All estimates took into account relevant survey sampling design and weighting using the *svy* command; for calculation of standard errors, strata with a single primary sampling unit were centered at the grand mean via *singleunit(centered)* specifications.

### Ethical review

NFHS-5 received ethical clearance from the ethical review board of the International Institute of Population Sciences (IIPS), Mumbai, India, and ICF USA. The secondary data analyses presented in this study utilized publicly available de-identified data and received a determination of Not Human Subjects Research from the Institutional Review Board of the University of California, San Diego (#810282).

## Results

### Sample demographics

Women included in the 12-month postpartum sample were 24.7 years old on average at the time of birth (standard deviation (SD) 4.7, range 12–48), and 11.7% (95% confidence interval (CI) 11.4–11.9%) of the sample were adolescents age 15–19. Average age of (first) cohabitation was 19.2 years (SD 3.6, range 10 to 45), with one in three respondents first cohabitating before age 18 (33.6%, 95% CI 33.2–34.0). About a fifth of women had no education (20.5%, 95% CI 20.2–20.9%), 12.1% (95% CI 11.9–12.4%) had primary education, 50.9% (95% CI 50.4–51.3%) had secondary education, and 16.5% (95% CI 16.1–16.9%) had tertiary education. Most women lived in rural areas (71.8%, 95% CI 71.3–72.3%).

About one-third of births included in the sample were first births (35.3%, 95% CI 35.0–35.7%), another third were second births (35.7%, 95% CI 35.4–36.0%), 16.2% were third births (95% CI 16.0–16.5%), and 12.7% were fourth or higher order births (95% CI 12.5–13.0%). Few births in the sample were multiples (twins or triplets, 0.9%, 95% CI 0.9–1.0%). Births were more likely to be male (53.4%, 95% CI 53.0–53.7%) than female (46.6%, 95% CI 46.3–47.0%). The majority of women had attended four or more ANC visits prior to birth (58.7%, 95% CI 58.2–59.1%), and had delivered in a facility (89.5%, 95% CI 89.2–89.8%).

Compared to the married WRA population not included in the postpartum sample, women in the postpartum sample were younger at time of birth (average age 24.7 vs 35.9 years, p < 0.001), had lower parity at time of birth (average 2.2 vs 2.3 births ever, p < 0.001), were more likely to have secondary or higher education (67% vs 55%, p < 0.001), and were more likely to live in rural areas (72% vs 67%, p < 0.001).

### PPC use, any method, overall and by month

Within the first 12 months postpartum, 59.2% (95% CI 58.8–59.7%) of women reported using any method of contraception.

**Fig. 1:**
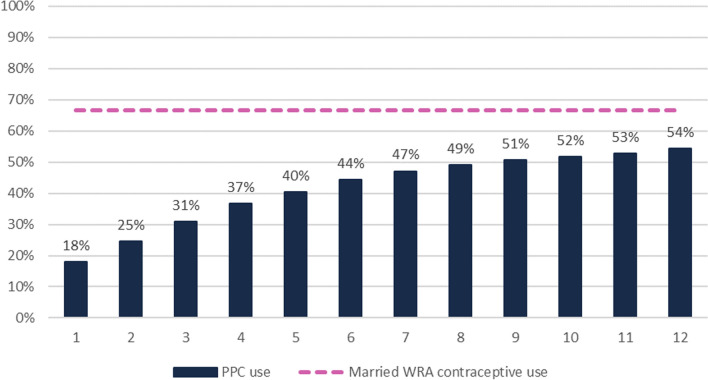
PPC use by month postpartum, any method

FP use increased by month postpartum, from 18.0% in month 1 postpartum (95% CI 17.7–18.3%) to 54.3% by month 12 postpartum (95% CI 53.9–54.7%) (Fig. 1). This is still well below the overall rate of contraceptive use for married WRA generally, 66.7% (95% CI 66.5–67.0%). Contraceptive use increased more rapidly from months 1 to 7 postpartum, with increases of 4% to 7% each month, and more slowly from months 7 to 12 postpartum, with monthly increases of less than 2% per month.

**Fig. 2:**
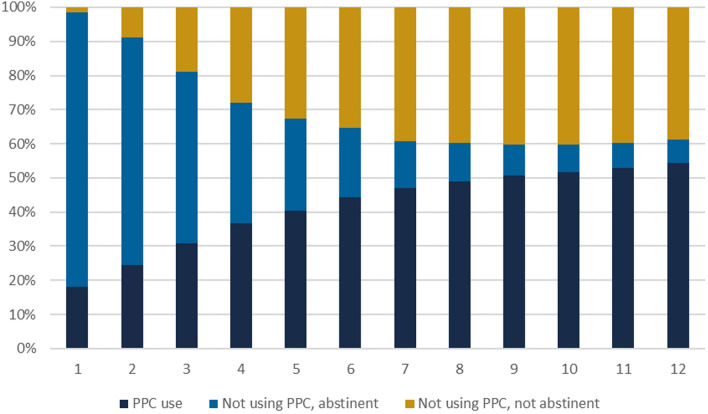
PPC use and continuous abstinence since birth, by month postpartum

Among non-users of PPC at 1 month, 98.1% (95% CI 98.0–98.3%) were abstinent; this decreased to only 15.1% (95% CI 14.7–15.6%) of non-users by month 12 (Fig. 2). Conversely, sexually active women who were not using any form of contraception increased from 1.5% (95% CI 1.4–1.6%) of the total postpartum population at month 1 to 38.8% (95% CI 38.4–39.2%) of the total postpartum population at month 12.

Among all postpartum women, 42.4% (95% CI 42.1–42.8%) were either abstinent or using a contraceptive method for the full 12-month postpartum time period; conversely, 57.6% (95% CI 57.2–57.9%) of women had at least one month of non-abstinence and non-use of contraception or a subsequent pregnancy within the postpartum period.

### PPC use, by specific method, overall and by month

Over the full 12-month postpartum time period, female sterilization and condoms were the most frequently utilized contraceptive methods, used by 15.1% (95% CI 14.9–15.4%) and 16.7% (95% CI 16.4–17.0%) of women, respectively at some point in the 12 months postpartum (Table 1).

**Table 1:** PPC use by specific method, overall and cross-sectionally by month postpartum

	Use at any month postpartum (%)	1 (%)	2 (%)	3 (%)	4 (%)	5 (%)	6 (%)	7 (%)	8 (%)	9 (%)	10 (%)	11 (%)	12 (%)
*Any method*	*59.2*	*18.0*	*24.5*	*30.9*	*36.7*	*40.4*	*44.3*	*47.1*	*49.1*	*50.6*	*51.8*	*52.8*	*54.3*
Female sterilization	15.1	8.7	10.0	11.1	11.8	12.5	13.2	13.7	14.1	14.6	15.0	15.4	16.0
IUD	4.1	2.6	2.8	2.9	3.0	3.1	3.2	3.3	3.3	3.4	3.4	3.4	3.5
Pill	8.2	0.8	1.6	2.4	3.2	3.8	4.6	5.4	5.8	6.0	6.2	6.4	6.7
Injectables	1.0	0.1	0.2	0.3	0.4	0.5	0.5	0.6	0.6	0.7	0.7	0.7	0.7
Condom	16.7	1.9	4.1	6.2	8.3	9.4	10.6	11.8	12.2	12.6	12.8	13.0	13.2
Rhythm	10.6	1.4	2.7	4.1	5.4	6.1	6.8	7.6	7.9	8.0	8.2	8.3	8.5
Withdrawal	6.8	0.9	1.6	2.4	3.2	3.7	4.2	4.7	5.0	5.2	5.3	5.4	5.5
LAM	2.0	1.5	1.5	1.5	1.4	1.2	1.1	–	–	–	–	–	–
Other methods^a^	0.3	0.0	0.1	0.1	0.1	0.1	0.1	0.2	0.2	0.2	0.2	0.2	0.2

^a^Other methods include: male sterilization, female condom, diaphragm, foam or jelly, 'other modern', or 'other traditional' methods; All were used by<0.01% of women in the 12 months postpartum

The use of various PPC methods differed substantially by month (Table 1). Female sterilization was the most popular contraceptive method in the first month postpartum, and remained the most popular method within each subsequent month. At month 1 postpartum, IUD was the second most popular method, but condoms became the second most utilized method for all subsequent months. Only LAM saw decreasing use over time, necessarily due to the time-limited nature of the method. All other methods had absolute increases in use over the 12 months postpartum.

**Fig. 3:**
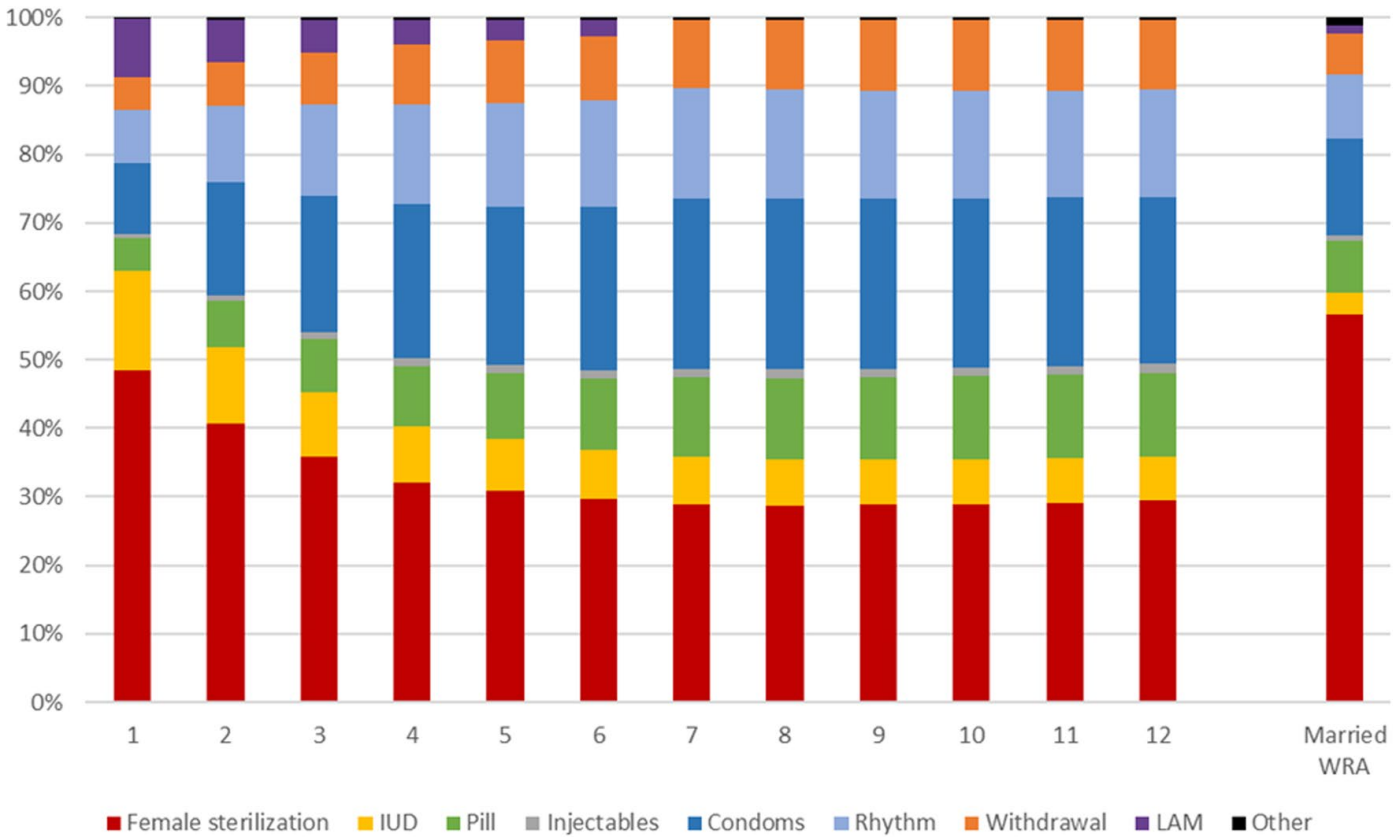
Method mix by month postpartum among PPC users and method mix among general WRA population

Just as absolute rates of specific method use varied over time, the relative use of specific methods—also known as the method mix—varied during the postpartum time period (Fig. 3). Specifically, female sterilization and IUDs had a larger share of method mix in month 1 postpartum (48.4% and 14.6%, respectively), decreasing until month 7 postpartum (29.0% and 6.9%, respectively). Conversely, the contribution of condoms, pills, withdrawal, and rhythm methods increased over those first 7 months. From month 7 to month 12 postpartum, there was little change in the method mix. At month 12 postpartum, the method mix varied notably from the population of married WRA as a whole. This was driven by a difference in the utilization of female sterilization, which comprised 29.4% (95% CI 28.9–29.9%) of method mix among 12 months postpartum women, and 56.8% (95% CI 56.5–57.1%) of the method mix among married WRA; this is likely driven by the older age and higher parity of the married WRA population.

### PPC initiation

**Fig. 4:**
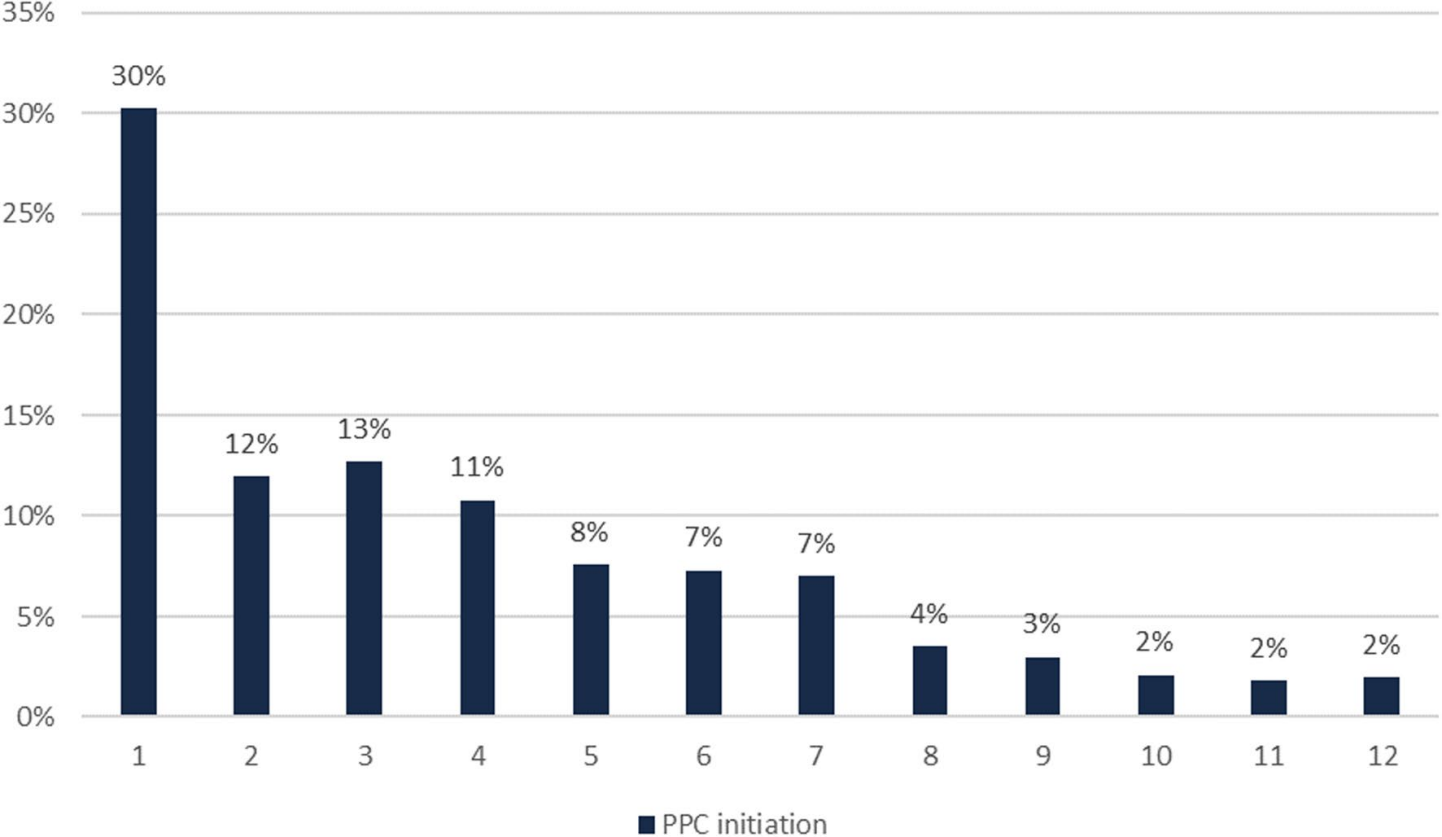
Percent of women first initiating PPC use by month, among all PPC users

Women who used PPC most frequently initiated that use in the first month postpartum (30.3% of PPC users first reported contraceptive use in month 1 postpartum, 95% CI 29.9–30.8%) (Table 2, Fig. 4). On average, women initiated contraceptive use 3.9 months after birth (95% CI 3.8–3.9). Initiation differed substantially by method. Initiation within the first month after birth was most common for female sterilization (57.5%), IUD (63.1%), and LAM users (78.0%), while all other method users were most likely to initiate three to five months after birth. By six months postpartum, 80.7% of all PPC users (95% CI 80.3–81.1%) had initiated contraceptive use. This rate was highest for those who used female sterilization (85.7%) and IUD (87.3%).

**Table 2:** PPC initiation, 6-month continuation, stopping contraceptive use, method switching, and subsequent pregnancy, overall and by specific method of contraception

	Mean month of initiation	% initiating in first 1mo postpartum	% initiating in first 6mo postpartum	% continuing use for at least 6mo postpartum^a^	% stopping any contraceptive use (at least 1 month)	% switching to another method^b^	% with subsequent pregnancy
*Any method*	*3.85*	*30.3*	*80.7*	*83.0*	*18.9*	*9.2*	*2.0*
Female sterilization	2.93	57.5	85.7	100	0	0	0
*Reversible methods*	*4.23*	*20.6*	*78.3*	*77.0*	*24.8*	*12.0*	*2.6*
IUD	2.65	63.1	87.3	83.6	15.4	9.7	1.9
Pill	5.26	11.3	66.7	71.2	21.1	11.5	1.6
Injectables	5.35	10.3	64.9	60.8	30.4	15.6	1.4
Condom	4.64	11.2	75.2	69.4	25.8	9.5	2.7
Rhythm	4.71	13.0	73.7	73.8	21.0	9.7	3.7
Withdrawal	4.93	12.4	70.9	76.5	16.4	10.7	2.7
LAM	1.48	78.0	100	39.3	76.6	51.9	2.0

^a^Among those who initiated within first 6 months postpartum

^b^Includes those who discontinued for 1 + month and then used an alternate method

### PPC continuation

Among women who initiated contraceptive use within the first 6 months postpartum, 83.0% (95% CI 82.6–83.5%) used contraception continuously for 6 months or more. This rate was by definition 100% for female sterilization users; for all reversible method users, 77.0% continued use for at least 6 months (95% CI 76.4–77.6%). Among reversible method users, 6-month continuation was highest for IUD (83.6%, 95% CI 82.1–85.0%) and (with the exception of LAM) lowest for injectable contraception (60.8%, 95% CI 56.6–64.8%).

Six-month continuation rates differed significantly by month of initiation for the most common reversible methods. For IUD, pill, injectable, condom, and rhythm method postpartum users, 6-month continuation rates were significantly higher among those initiating within 1 month after birth compared to those initiating 2 or 3 months after birth (p<0.05) (Table 3).

**Table 3:** Percent of women continuously using PPC method for 6 or more months by postpartum month of initiation, among select method PPC users within first 6 months after birth

	Postpartum month of initiation					
	1 (%)	2 (%)	3 (%)	4 (%)	5 (%)	6 (%)
IUD	86.4	70.0^a^	76.6^a^	79.7^a^	80.0	79.4
Pill	74.9	60.2^a^	63.3^a^	71.4	75.7	82.9^b^
Injectable	80.0	52.7^a^	59.2^a^	47.6^a^	60.3^a^	71.6
Condom	74.8	62.5^a^	67.0^a^	67.4^a^	71.1^a^	79.4^b^
Rhythm	81.4	69.3^a^	68.7^a^	72.4^a^	74.4^a^	80.8
Withdrawal	77.5	73.0	73.3	74.5	78.5	84.8^b^

^a^Statistically significantly (p < 0.05) lower continuation relative to month 1, adjusted Wald test

^b^Statistically significantly (p < 0.05) higher continuation relative to month 1, adjusted Wald test

### PPC stopping

Among all PPC users, 18.9% (95% CI 18.5–19.3%) stopped using contraception entirely for at least one month after initiation (Table 2). Among PPC users who first used a reversible method, 24.8% (95% CI 24.3–25.4%) stopped using contraception entirely for at least one month. With the exception of LAM users, stopping use of any contraception for at least one month was most common among users of injectable contraception (30.4%, 95% CI 27.4%-33.7%) and condoms (25.8%, 95% CI 25.0–26.6%).

### PPC method switching

Among all PPC users, 9.2% (95% CI 8.9–9.5%) used more than one method within 12 months postpartum (e.g. switched method at least once) (Table 2). Among PPC users who first used a reversible method, 12.0% (95% CI 11.6–12.4%) switched methods at least once in the 12 months after birth; 10.5% (95% CI 10.2–10.8%) switched methods once, while 1.5% (95% CI 1.4–1.6%) switched methods twice or more. Of the women who switched methods, 59.8% (95% CI 58.4–61.2%) switched directly to another method with no months of non-use, while 40.2% (95% CI 38.8–41.6%) had at least one month of non-use before using a different method. LAM users, by nature of the time-limited nature of method use, were most likely to switch to another method; 51.9% (95% CI 49.7–54.2%) of LAM users used another method by month 12 postpartum.

**Fig. 5:**
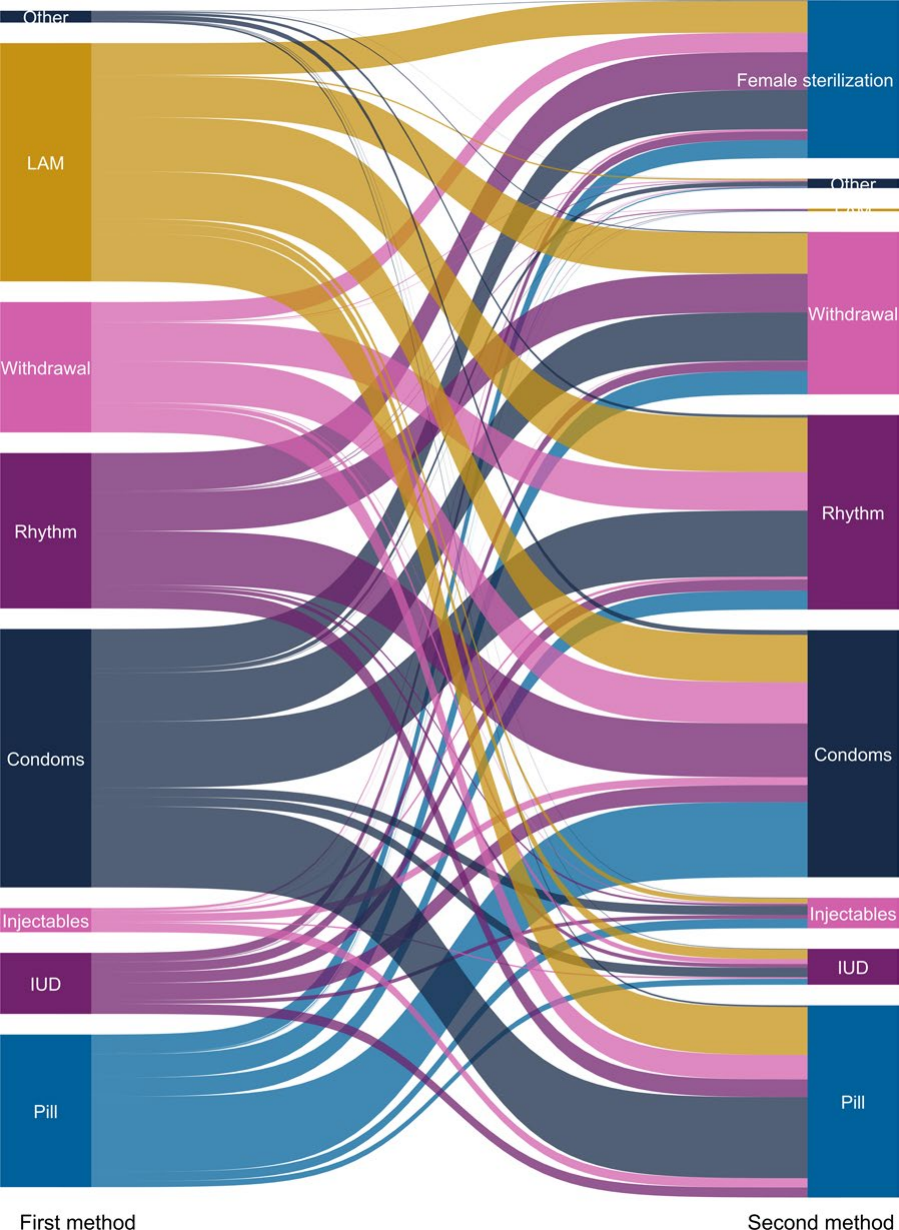
Contraceptive method switching within 12 months postpartum, among women who switched methods

Among women who switched contraceptive methods within 12 months postpartum, they were most commonly switching *from* using condoms—26.7% (95% CI 25.4–28.0%) of women who switched methods first used condoms (Fig. 5). These condom users most commonly switched *to* using pills (31.0%, 95% CI 28.4–33.6%) or rhythm method (28.0%, 95% CI 25.6–30.6%). Women also commonly switched *to* using condoms—24.6% (95% CI 23.4–25.8%) of women who switched methods switched to condoms. These women were *also* most commonly switching *from* using pills (31.2%, 95% CI 28.4–34.2%) or rhythm method (24.7%, 95% CI 22.2–27.2%).

### Subsequent pregnancy in postpartum time period

In the 12 months postpartum, 5.2% (95% CI 5.0–5.4%) of women reported a subsequent pregnancy. Among women who had a subsequent pregnancy within 12 months, 22.5% (95% CI 21.3–23.8%) used contraception at some point before the pregnancy, and 12.8% (95% CI 11.9–13.8%) were using a contraceptive method in the month immediately preceding the pregnancy. Of the women who indicated method use in the month prior to pregnancy, 39.6% (35.7–43.6%) indicated method failure; these women were most likely to report using condoms, rhythm, or withdrawal methods.

### Reasons for PPC discontinuation

Women who discontinued using a method of PPC, whether they subsequently used no method, switched methods, or became pregnant, provided detail on why they discontinued using the method in the discontinuation calendar. Women most frequently cited method-related reasons, including side effects, inconvenience or lack of privacy to use, or that they ‘did not like method’ (18.9%, 95% CI 18.2–19.7%) (Table 4). Other fertility-related reasons included infrequent sex, menopause, and marital dissolution, while other non-fertility-related reasons included access, availability, cost, and fatalism. Reasons for discontinuation differed meaningfully by method. Reasons also differed by the nature of discontinuation, e.g. the contraceptive use status in the month following discontinuation (use of no method, use of a different method, or pregnancy). Those who stopped using any method most frequently reported ‘other fertility-related’ reasons, those who switched methods most frequently reported method-related reasons, and those with a subsequent pregnancy most frequently reported method failure as the reason for discontinuing their method.

**Table 4:** Reasons for PPC discontinuation, overall, by method, and by nature of discontinuation

	Method failure (%)	Wanted to become pregnant (%)	Method related reasons (%)	Wanted more effective method (%)	Husband opposed (%)	Other fertility-related reasons (%)	Other non-fertility-related reasons (%)
**Method**							
*Any method*	*6.2*	*15.5*	*18.9*	*12.2*	*16.4*	*17.4*	*13.3*
IUD	6.3	11.3	57.8	8.2	4.5	3.8	8.2
Pill	4.2	15.0	28.4	7.8	15.8	13.4	15.4
Injectable	6.2	14.0	24.7	10.4	12.8	17.3	14.7
Condom	5.5	17.8	16.2	6.8	19.7	18.7	15.3
Rhythm	7.8	15.7	8.6	8.7	20.7	24.5	14.0
Withdrawal	7.9	16.8	13.4	12.2	20.4	21.3	8.0
LAM	3.8	3.9	15.7	47.7	5.8	10.8	12.3
**Nature of discontinuation**							
Stopped use of any method	4.8	16.9	17.9	6.4	18.4	20.7	14.9
Switched method	3.0	7.4	24.5	23.6	18.8	11.7	10.9
Subsequent pregnancy	39.6	29.2	6.6	3.2	6.0	9.6	5.9

### PPC use among adolescents age 15–19

**Fig. 6:**
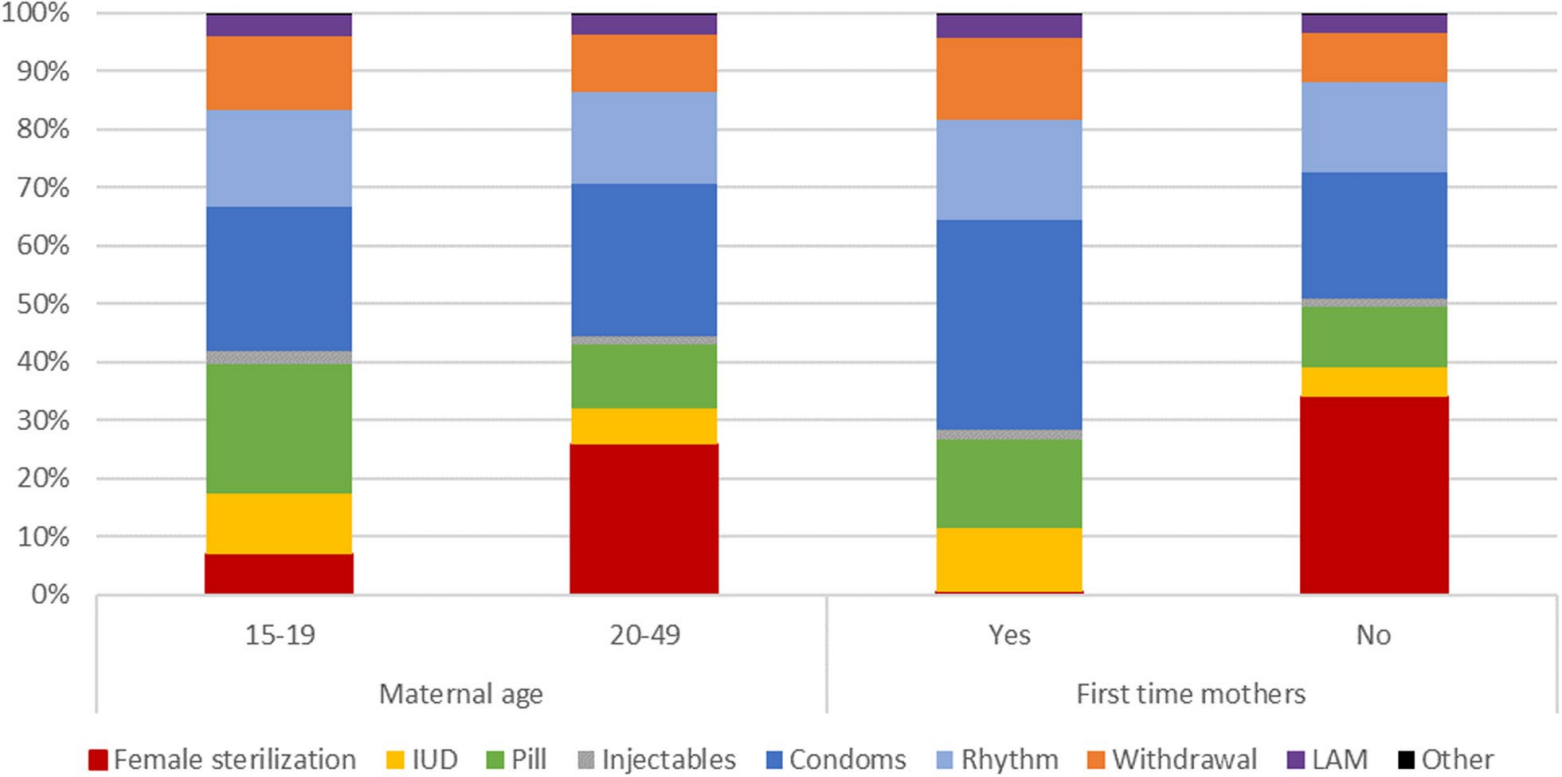
PPC method mix among PPC users*, by maternal age and first-time parent status. *First method used postpartum, if multiple methods used (9.2% of all PPC users used > 1 method)

The postpartum sample analyzed here included 11.7% (95% CI 11.4–11.9%) adolescent mothers (age 15–19) at time of birth. Adolescent mothers were significantly less likely to use PPC compared to mothers age 20–49 (48.6% vs 60.6%, p < 0.001). This is driven largely by differences in female sterilization use; only 3.6% of adolescent mothers used postpartum female sterilization, compared to 16.7% of mothers age 20–49 (p < 0.001). Use of any reversible PPC method during the postpartum period was comparable between adolescent mothers and those age 20–49 (45.2% vs 45.0%, p = 0.71). Adolescent mothers were more likely to use pills (12.3% vs 7.6%, p < 0.001) and IUDs (5.2% vs 4.0%, p < 0.001). Conversely, adolescent mothers were less likely to use condoms (13.1% vs 17.2%, p < 0.001) and rhythm method (9.1% vs 10.8%, p < 0.001); use of injectables (1.2% vs 1.0%, p = 0.19), withdrawal method (7.2% vs 6.8%, p = 0.12), and LAM (1.8% vs 2.0%, p = 0.11) did not differ significantly by maternal age. The relative PPC method mix of mothers age 15–19 and 20–49 is shown in Fig. 6. Adolescent mothers were significantly more likely to have a subsequent pregnancy in the 12 months postpartum compared to mothers age 20–49 (8.7% vs 4.7%, p < 0.001).

Among pill users, mothers age 15–19 were equally likely as mothers age 20–49 to initiate use in the first month (12.4% vs 11.7%, p = 0.61) or within the first 6 months postpartum (69.1% vs 70.7%, p = 0.37). Adolescent mothers who used pills within the first 6 months postpartum were more likely to continue pill use for at least 6 months (77.3% vs 69.5%, p < 0.001). Adolescent mothers who used pills were less likely to discontinue use of any PPC (19.6% vs 22.9%, p = 0.04) and less likely to switch to another method (8.4% vs 12.1%, p = 0.003), compared to mothers age 20–49.

Among IUD users, mothers age 15–19 were more likely than mothers age 20–49 to initiate use in the first month postpartum (72.1% vs 64.3%, p = 0.003), though were equally likely to initiate within the first 6 months postpartum (90.4% vs 88.8%, p = 0.42). Adolescent mothers who used an IUD within the first 6 months postpartum were equally likely to continue IUD use for at least 6 months (83.4% vs 83.6%, p = 0.95), Adolescent mothers who used an IUD were also equally likely to discontinue use of any PPC (16.8% vs 15.9%, p = 0.66) and to switch to another method (9.4% vs 9.7%, p = 0.84), compared to mothers age 20–49.

Among condom users, mothers age 15–19 were slightly more likely than mothers age 20–49 to initiate use in the first month postpartum (14.3% vs 12.0%, p = 0.04), though were equally likely to initiate within the first 6 months postpartum (78.1% vs 78.0%, p = 0.94). Adolescent mothers who used condoms within the first 6 months postpartum were equally likely to continue condom use for at least 6 months compared to mothers age 20–49 (66.5% vs 69.5%, p = 0.10). Adolescent mothers who used condoms were more likely to discontinue use of any PPC (29.6% vs 26.4%, p = 0.03), and were equally likely to switch to another method (10.6% vs 9.3%, p = 0.25).

### PPC use among first-time mothers

The postpartum sample analyzed here included 35.3% (95% CI 35.0–35.7%) first-time mothers (birth order / parity = 1), average age 22.3 years. First-time mothers were significantly less likely to use PPC compared to women who had previously given birth (50.5% vs 64.0%, p < 0.001). This is again driven largely by differences in female sterilization use; only 0.3% of first-time mothers used postpartum female sterilization, compared to 23.3% of women with previous births (p < 0.001). Conversely, first-time mothers were more likely to use condoms (19.6% vs 15.1% p < 0.001), IUDs (5.7% vs 3.3%, p < 0.001), pills (9.0% vs 7.7%, p < 0.001), and withdrawal method (8.2% vs 6.1%, p < 0.001) (Fig. 6). First-time mothers were slightly less likely to use injectable contraception (0.9% vs 1.1%, p = 0.01) and rhythm method (9.9% vs 11.0%, p < 0.001), and there was no difference in LAM use (2.0% vs 2.0%, p = 0.80), compared to women who had previously given birth. First-time mothers were more likely to have a subsequent pregnancy in the 12-month postpartum time period (8.4% vs 3.4%, p < 0.001).

Among pill users, first-time mothers were less likely than mothers who had previously given birth to initiate contraceptive use in the first month postpartum (10.2% vs 12.8%, p = 0.02) or within the first 6 months postpartum (66.9% vs 72.6%, p < 0.001). First-time mothers who used pills within the first 6 months postpartum were similarly likely to continuously use pills for at least 6 months (72.8% vs 69.7%, p = 0.053). First-time mothers who used pills were similarly likely to discontinue using any form of PPC for at least one month (21.3% vs 23.0%, p = 0.12), compared to women who had previously given birth, and were less likely to switch methods (9.6% vs 12.7%, p < 0.001).

Among IUD users, first-time mothers were more likely than women who had previously given birth to initiate use in the first month postpartum (68.2% vs 62.8%, p = 0.003), though were equally likely to initiate within the first 6 months postpartum (89.2% vs 88.9%, p = 0.81). First-time mothers who used an IUD within the first 6 months postpartum were similarly likely to continuously use IUDs for at least 6 months (82.9% vs 84.1%, p = 0.41). First-time mothers who used an IUD were more likely to discontinue using any form of PPC for at least one month (17.9% vs 14.3%, p = 0.02), and less likely to switch methods (7.8% vs 11.5%, p < 0.001), compared to women who had previously given birth.

Among condom users, first-time mothers were less likely than mothers who had previously given birth to initiate use in the first month postpartum (11.9% vs 13.1%, p < 0.001) or within the first 6 months postpartum (75.4% vs 79.9%, p < 0.001). First-time mothers who used condoms within the first 6 months postpartum were more likely to continuously use condoms for at least 6 months (70.7% vs 68.3%, p = 0.02). First-time mothers who used condoms were similarly likely to discontinue using any form of PPC for at least one month (26.2% vs 27.0%, p = 0.27) compared to women who had previously given birth, and were less likely to switch methods (7.6% vs 10.8%, p < 0.001).

### PPC use by sociodemographic and birth characteristics

PPC use differed meaningfully by most examined sociodemographic and birth factors in bivariate comparisons, both overall and for specific method use (Annex Table S1).

**Fig. 7:**
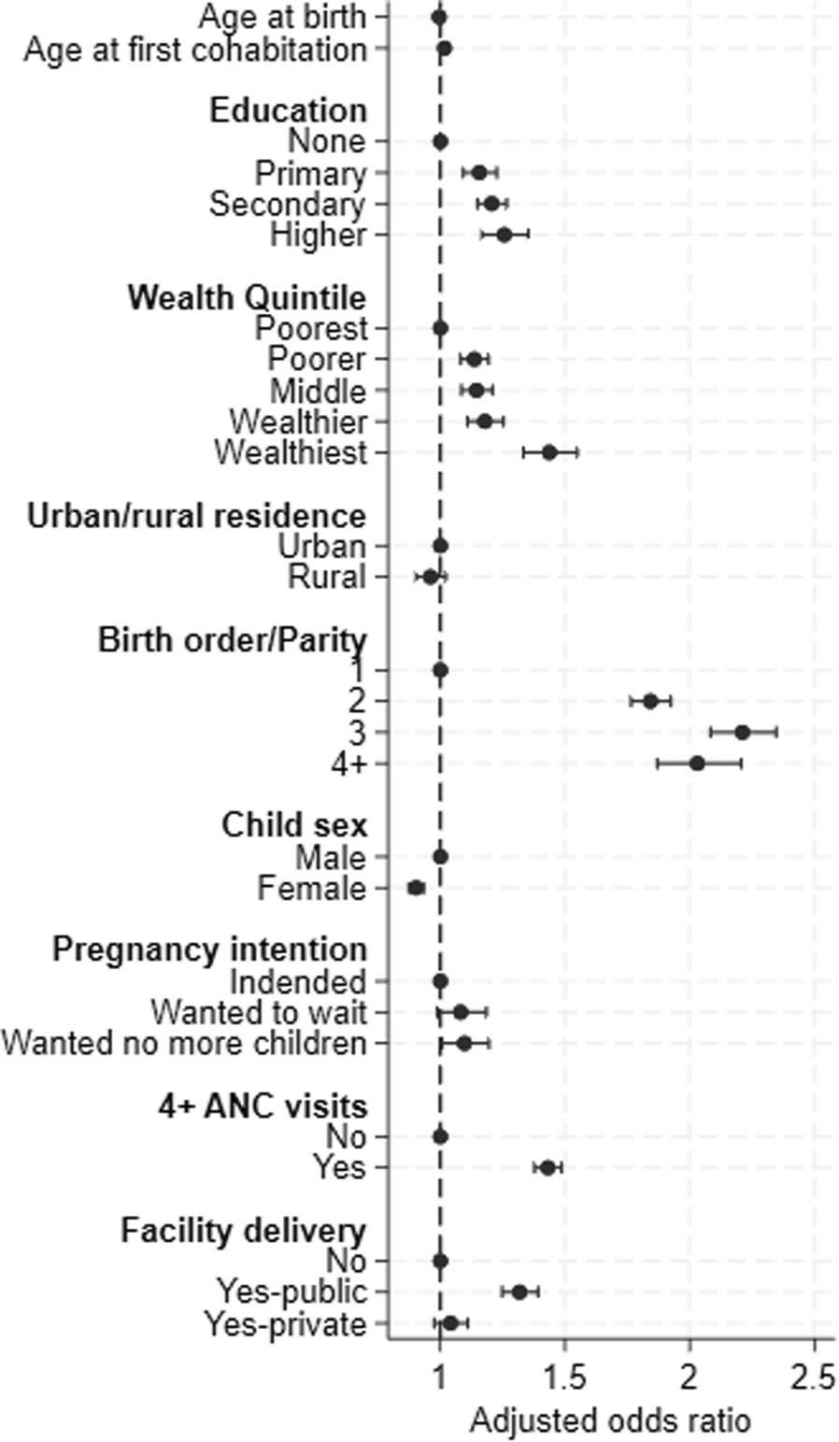
Adjusted logistic regression model estimates, any PPC use

In adjusted models, most sociodemographic and birth factors were associated with overall PPC use (Fig. 7, Annex Table S2). Older age of first cohabitation, higher education, higher wealth quintile, higher parity, male child, unintended pregnancy, 4 + ANC visit attendance, and public facility delivery were all associated with significantly greater uptake of PPC; age at birth and urban vs rural residence were not significantly associated with PPC overall.

**Fig. 8:**
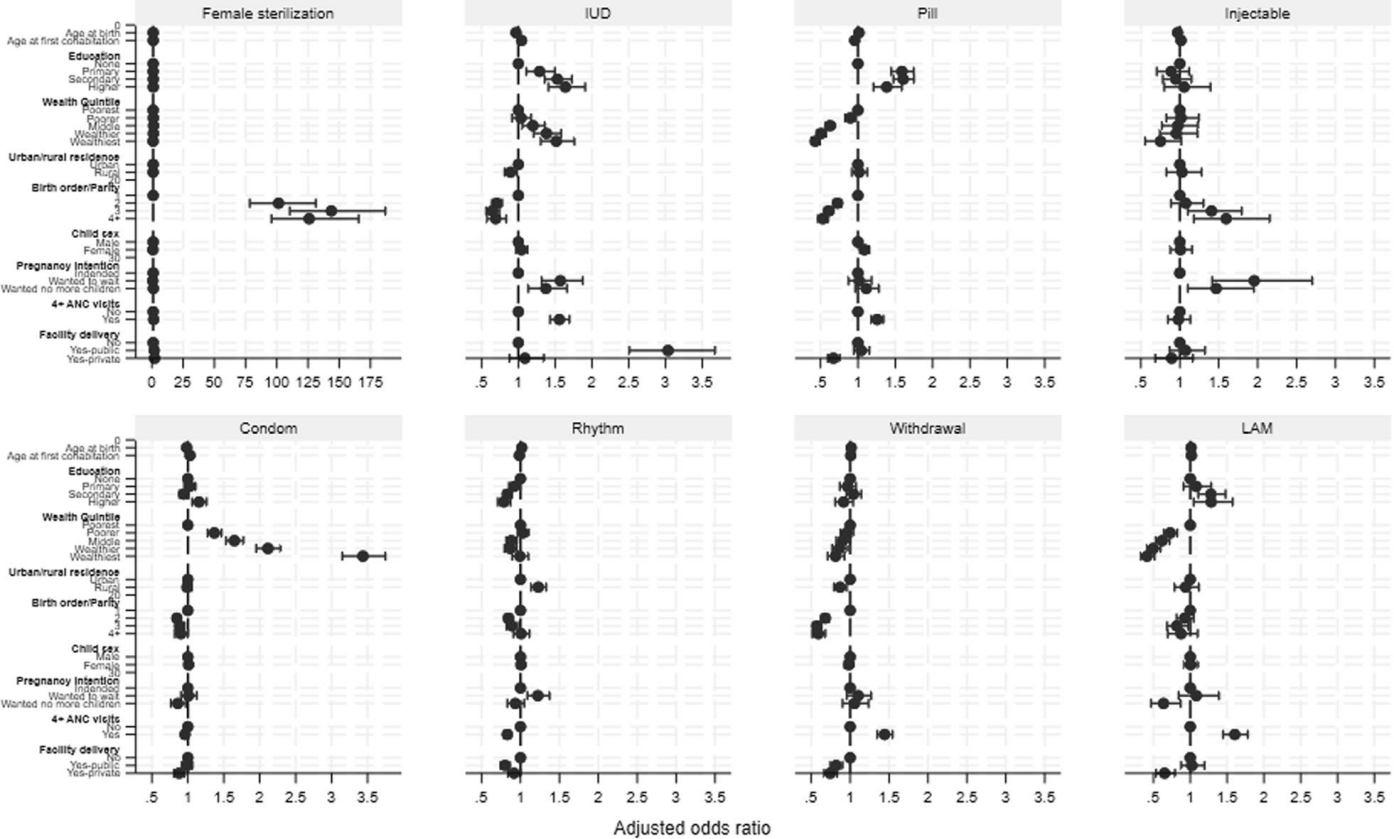
Adjusted logistic regression model estimates, by specific PPC method

Patterns of association between sociodemographic factors and PPC use differed by specific method (Fig. 8, Annex Table S2). Older women were more likely to use pills, rhythm, and withdrawal, while younger women were more likely to use female sterilization, IUD, injectables, and condoms. Women married at younger age were more likely to use pills and rhythm, and less likely to use female sterilization, IUD, and condoms. Women with higher levels of education were more likely to use female sterilization, IUD, and pills, and less likely to use rhythm method. Women in the higher wealth quintiles were more likely to use IUD and condoms, and less likely to use pills, withdrawal, and LAM methods. Women in urban areas were more likely to use IUD and withdrawal methods, while women in rural areas were more likely to use rhythm method.

Patterns of association between birth characteristics and PPC use also differed by specific method. Women who gave birth to a male child were more likely to use female sterilization and less likely to use pills. Women who indicated the pregnancy was not intended were more likely to use female sterilization, IUD, and injectables, and less likely to use condoms and LAM. Higher birth order was associated with greater use of female sterilization and injectables, and lower use of IUD, pills, and withdrawal methods. Measure of healthcare system engagement—4 + ANC visits and facility delivery – were associated with significantly greater uptake of female sterilization and significantly lower uptake of withdrawal method. Relative to non-facility delivery, delivery in a public facility was additionally associated with significantly greater uptake of IUD and lower uptake of rhythm method, and delivery in a private facility was associated with significantly lower uptake of pill, condom, and LAM method use.

### Sensitivity analyses: 24-month postpartum time frame

Overall PPC use was similar when examining a 24-month rather than 12-month postpartum time frame.

Using a 24-month time frame, 60.8% of women (95% CI 60.4–61.3%) used a method of contraception at any point in the first two years postpartum, and 54.9% of all women (95% CI 54.5–55.4%) initiated PPC by 12 months (Annex Table S3). This suggests that 5.9% of all women first initiated PPC use more than one year postpartum. Women who initiated PPC between 12 and 24 months postpartum were most likely to use female sterilization (25.8%), condoms (23.9%), or rhythm method (17.9%).

Initiation of PPC in the 24-month sample was later on average, and less likely to occur in the first 1 or 6 months postpartum, by nature of the longer follow-up time period definition (Annex Table S4). Of all PPC use within 24 months, 90.3% (95% CI 90.0–90.6%) was initiated within the first 12 months. Outside of LAM, postpartum IUD users were most likely to initiate within 12 months (90.1%), while injectable users were least likely to initiate within 12 months (71.4%). Six-month PPC continuation rates, among those who initiated use within the first 6 months postpartum, were high and nearly equivalent in the 24-month sample (81.9%, 95% CI 81.4–82.3%). Twelve-month PPC continuation rates, among those who initiated use within the first year, were slightly lower at 74.1% (95% CI 73.6–74.6%). Rates of stopping PPC use entirely for at least 1 month after initiation were higher in the 24-month sample compared to the 12-month sample, with 27.4% of women (95% CI 26.9–27.9%) stopping PPC use before month 24. Similarly, rates of method switching were also higher in the 24-month sample; 13.5% of women (95% CI 13.1–13.8%) and 17.8% of reversible method users (95% CI 17.3–18.3%) switched PPC methods at least once. Among PPC users who first used a reversible method, 82.2% (95% CI 81.7–82.7%) used one method, 15.7% (95% CI 15.2–16.1%) used two methods, and 2.1% (95% CI 2.0–2.3%) used three or more methods by 24 months postpartum.

The rate of subsequent pregnancy was substantially higher when using a 24-month postpartum sample, where 23.1% (95% CI 22.8–23.5%) of women reported a subsequent pregnancy within 2 years of the reference birth. Additionally, 9.5% of women (95% CI 9.3–9.8%) in the 24-month postpartum sample became pregnant and had a subsequent birth within 24 months of the reference birth. Of these, 83.8% (95% 82.8–84.7%) indicated that it was intended at that time, 11.0% (95% CI 10.2–11.8%) indicated they wanted to get pregnant later, and 5.3% (95% CI 4.7–5.8) indicated they did not want to get pregnant again. Subsequent birth was more common among the subpopulations of interest, adolescents (16.6% among mothers age 15–19 vs 8.5% among mothers age 20–49) and first-time mothers (14.7% among first-time mothers vs 6.5% among those who had already given birth). Reported birth intendedness was equivalent between first-time mothers and those who had already given birth, while older mothers were more likely than adolescent mothers to report that a subsequent birth within 24 months was intended.

Of women who had a subsequent birth, 11.3% (95% CI 10.6–12.1%) were using FP in the month immediately prior that pregnancy, most commonly rhythm (4.2%, 95% CI 3.8–4.7%), condoms (3.5%, 95% CI 3.1–3.9%) or withdrawal (2.2%, 95% CI 1.9–2.5%) methods. Those who were using FP immediately prior to a pregnancy which resulted in birth were more likely to report that the pregnancy was unintended (26.7% versus 15.0%, p < 0.001).

## Discussion

This paper presents a unique and nuanced overview of complex patterns of postpartum contraceptive use among married women in India. We find that 59% of women used a method of contraception at any point within the first year postpartum, and that condoms and female sterilization were the most commonly used PPC methods overall. However, patterns of PPC use differed substantially by month and by method. Though most women who initiated PPC use continued for at least six months, 9% of all PPC users subsequently switched methods within the first year postpartum, and 19% stopped using contraception entirely, suggesting substantial dynamism in contraceptive use during the postpartum period in India.

Though initiation of PPC use within the first month was common for sterilization and IUD, women initiated PPC use four months after birth on average, with one in five women first initiating contraception after six months postpartum. While many PPC programs focus on immediate or early postpartum provision [14], this analysis indicates that women in India regularly access PPC beyond the immediate postpartum window, and that programs serving postpartum women should consider a wider time frame for engagement and intervention. The significant volume of PPC uptake occurring in months four through seven, coupled with the meaningful increases in resumption of sexual activity and return of menses during those months, suggests this is a time when women recognize a need for and seek out contraception. Having available contraceptive services during this four to seven month postpartum time frame when women are ready to resume or initiate contraceptive use after birth is therefore critical, and includes person-centered contraceptive counseling, in-stock availability of a full range of methods, and appropriate providers and facilities for methods which require a procedure.

We find substantial change in contraceptive use overall and by method during the first year postpartum. Among women using reversible PPC methods, one in four stopped using contraception entirely for at least one month after initiation, and one in eight switched methods at least once. Initiation of PPC or measurement of PPC use at a single timepoint is therefore insufficient to assess and meet the contraceptive needs of postpartum women. A reduction in subsequent PPC stopping or switching due to dissatisfaction with or inability to use a method may be facilitated by greater use of high-quality person-centered contraceptive counseling to increase knowledge of and satisfaction with the method used based on the unique needs and circumstances of each woman [21, 50–52]. Though we summarize reasons for method discontinuation based on the pre-determined list available in NFHS, there remains substantial opportunity for greater understanding of the drivers and pathways by which women decide to stop using a method and/or choose an alternate one. Future research should explore the decision-making processes by which these changes in contraceptive use status occur, inclusive of women’s agency and desire to determine use within their partnership and household and with their healthcare provider.

Compared to the married WRA population generally, contraceptive use for postpartum women was lower overall but with a greater diversity of methods used [26]. More specifically, the prevalence of female sterilization use was much lower (15% among postpartum women vs 38% for the married WRA population generally), but reversible method use was much higher (45% among postpartum women vs 28% for the married WRA population generally). Postpartum women are therefore more likely to be interested in and to seek reversible contraceptive methods, and to require continued interaction with the health system to maintain desired contraceptive coverage. From a policy lens, this suggests that contraceptive counseling to postpartum women should therefore include all options, and practical availability of the range of methods should be ensured. In general, contraceptive method availability and cost regularly differ by health facility type, and the differences in PPC utilization of specific methods between women who delivered in public versus in private health facilities suggests this is likely also true for postpartum women specifically. Though most women do not receive PPC at the time of delivery, the location of delivery is a proxy for the nature of their healthcare access more generally. We saw, after controlling for other demographic characteristics also associated with method choice, that women who used public facilities were more likely to use IUDs and pills compared to women who delivered in a private facility. This greater use may be due in part to government subsidization of cost for these methods, or driven in part by provider pressure (conscious or not) to use these methods in the presence of method-specific initiatives and incentives. This suggests that differences in availability and cost across healthcare settings contributes to differences in observed utilization. To ensure truly patient-centered choice in PPC method use, availability and affordability of the full range of contraceptive options without coercion is a foundational requirement of family planning policy and programs.

We found that adolescent mothers were significantly less likely to use any form of PPC compared to mothers age 20–49, driven largely by lower female sterilization use. Conversely, we found that adolescent mothers were more likely to use pills and IUDs than their older counterparts, and we found significant variation in initiation, continuation, and method switching within specific method types. Additionally, adolescent mothers were more likely to have a subsequent pregnancy in the 12 months postpartum compared to mothers age 20–49. The unique contraceptive use patterns and needs of this population support the ongoing use of policies and programs tailored to adolescents, including the Adolescent Reproductive and Sexual Health Strategy (ARSH) and the National Adolescent Health Programme (Rashtriya Kishore Swaasthya Karyakram, RKSK) [53, 54]. RKSK explicitly recognizes the need for postpartum care and counseling to support delay of repeat pregnancy as strategic priorities [38], though more explicit integration of person-centered PPC counseling and access to desired methods could better support this population. From a policy perspective, adolescents should also continue to be recognized as a unique population in contraception and family planning initiatives and materials more broadly [55]. Additionally, while our analyses were limited to married individuals, unmarried adolescents face additional barriers to contraceptive access and utilization, and should be explicitly recognized in all adolescent-focused contraception strategies [56].

Approximately one third of our sample were first-time mothers; as with adolescent mothers, we found unique patterns of method use for this population. First-time mothers were significantly less likely to use any form of PPC compared to women who had given birth previously, though this differed by method use. First time mothers were less likely to use female sterilization and more likely to use condoms, pills, IUDs, and withdrawal than women who had previously given birth. Existing initiatives, such as FP kits for newlywed couples under Mission Parivar Vikas (MPV) [57], and public health messaging [58] largely focus on delaying first birth for young and newly-married couples. After the first birth has occurred, however, the data presented here suggest that first-time mothers present a unique population and still warrant targeted attention in the postpartum period. Many of these women may not have previously used contraception, in part due to fertility pressure to have children early in marriage, and may require additional contraceptive information and counselling compared to their higher-parity counterparts. PPC policies and programs should more explicitly recognize first-time mothers as a key population with unique vulnerabilities and needs.

Understanding the dynamic nature of postpartum contraceptive use is an important contribution to recent shifts in family planning programs and policies to deprioritize contraceptive use and uptake targets as measures of success, and instead focus on reproductive agency and empowerment [59–62]. Examining individual postpartum use pathways, rather than estimating aggregate levels of usage at a single timepoint and positioning that prevalence as a number representative of all women, is a meaningful step towards recognizing the natural complexity of contraceptive goals and agency, as well as the power dynamics and norms that influence those choices. As the field of family planning develops metrics that are more focused on agency and empowerment [41, 61–66], and shifts away from the oversimplified and terminologically imprecise paradigm of ‘supply’ and ‘demand’ [60, 67], revisiting the way that we conceptualize and measure PPC offers a key lens to provide family planning programs with more actionable data to support person-centered postpartum contraceptive choice and enable the achievement of self-determined reproductive goals.

### Strengths and limitations

This paper has a number of strengths, not least of which is use of the recently collected, nationally-representative NFHS-5 dataset, which allows us to disaggregate contraceptive calendar data by method, timing, and subpopulation. Our analysis of contraceptive calendar data enabled us to analyze month-by-month changes in contraceptive behavior at an individual level, creating a picture of dynamic change that is distinct from prior cross-sectional analyses. Our analytic approaches were robust, accounting for key sociodemographic characteristics in our regressions, and including a longer postpartum time window in our sensitivity analysis. This is one of the first studies that provides a comprehensive analysis of the dynamics of postpartum contraception, which has been an area that has not received adequate attention on the contraceptive user pathway. Nevertheless, these findings are subject to several limitations. First, the use of the retrospective contraceptive calendar data for births which occurred up to five years prior to survey is subject to meaningful recall bias, with greater likelihood of inaccuracy among those with more complex reproductive histories or older births [68, 69]. Secondly, the contraceptive calendar allows for reporting of only one method at a time, and only summarizes monthly use; concurrent use of multiple methods or use for partial months will not be captured. This also limits us from distinguishing between immediate postpartum contraceptive use (e.g. within 48 h of birth) vs use initiated later within the first month postpartum, the timing of which has important programmatic implications. Thirdly, this data represents births which occurred between July 2014 and April 2020, and the majority of data therefore reflects the time period before the COVID-19 pandemic and its resultant health system and societal impacts. National Health Mission Health Management Information System data suggest that there were significant reductions in provision of IUD, pill, and injectable contraceptives, along with a decline in institutional deliveries and other related maternal and child health services, particularly in the first year of the COVID-19 pandemic [70]. Our estimates of PPC use may therefore overestimate levels of use for 2020–2021, when access to contraceptive and family planning services was more limited.

Finally, though we report several outcomes along the continuum of contraceptive use, we do not report any measures of accurate contraceptive knowledge, access, satisfaction, or agency due to limited measure availability within the DHS, particularly for retrospective calendar data. Though we report reasons for method discontinuation which include dissatisfaction with a method, women may be dissatisfied but continue use (most extremely in the case of sterilization regret) [71]. We also cannot ascertain from the data available whether non-use of PPC is the result of dissatisfaction with available methods, a lack of knowledge or agency to use contraception, or other reasons, and we cannot determine whether women who did use PPC were able to choose to use a desired method based on complete and accurate information and freedom from coercion. These data limitations underscore the need for increased person-centered measurement in this space and a focus on contraceptive outcomes beyond simply use or met need [60, 67].

## Conclusion

In this study of postpartum contraceptive use in India, we find that contraceptive utilization varies widely by month, by method, and by subpopulation. We present evidence of increasing uptake over the first year postpartum, though postpartum contraceptive use is consistently lower than contraceptive use among women of reproductive age; this difference is largely driven by lower utilization of female sterilization in postpartum women. Reversible methods are disproportionally represented in the method mix of postpartum women in India, allowing for distinct and multifaceted patterns of use throughout the postpartum period, including substantial rates of stopping use, switching methods, and subsequent pregnancy. We also find meaningful differences in postpartum contraceptive method mix and utilization patterns for adolescent mothers and first-time mothers when compared to their counterparts. The postpartum period is a unique window of opportunity in which to support women’s ability to utilize their desired contraceptive methods. Promoting reproductive agency and ensuring contraceptive access can enable women to delay or prevent a subsequent pregnancy and meet their health, fertility and broader personal goals. These results collectively suggest the need for conceptualizing contraceptive use in the postpartum period as a dynamic process and reinforce growing calls for a focus on agency- and empowerment-centered contraceptive measures that better represent the continuum of contraceptive use experienced over the life course.

## Supplementary Information

Additional file 1: Annex Document 1. doc contains NFHS-5 contraceptive method descriptions.

Additional file 2: Annex Tables.xlsx contains the following 4 additional tables: Annex Table 1: Sociodemographic and birth characteristics associated with PPC method use, bivariate comparisons; Annex Table 2: Sociodemographic and birth characteristics associated with PPC method use, multivariate comparisons; Annex Table 3: PPC use by specific method, overall and cross-sectionally by month postpartum, 24-month sample sensitivity analysis; Annex Table 4: PPC initiation, 6-month continuation, stopping contraceptive use, method switching, and subsequent pregnancy, overall and by specific method of contraception, 24-month sample sensitivity analysis.

## Data Availability

The data used in this study are publicly available from the Demographic and Health Surveys Program website at https://dhsprogram.com/data/. Registration is required to request access to the datasets; registration can be completed here: https://dhsprogram.com/data/new-user-registration.cfm. All analytic code used in this study is available upon request from the corresponding author.

## Abbreviations

AOR: Adjusted odds ratio
ARSH: Adolescent Reproductive and Sexual Health Strategy
CI: Confidence interval
FP: Family planning
IUD: Intrauterine device
LAM: Lactational Amenorrhea
LMIC: Low- and middle-income country
MPV: Mission Parivar Vikas
NFHS: National Family and Health Survey
PPC: Postpartum contraception
PSU: Primary sampling unit
RKSK: Rashtriya Kishore Swaasthya Karyakram
SD: Standard deviation
WRA: Women of reproductive age
