# Supplementary material for: The state of postpartum contraceptive use in India: descriptive lessons from nationally representative survey data
Source: Reprod Health. 2025 Mar 13;22:39. doi: 10.1186/s12978-025-01978-3 (PMC11905474; doi:10.1186/s12978-025-01978-3)
Supplement: Supplementary file 1 — Additional file 1: Annex Document 1. doc contains NFHS-5 contraceptive method descriptions. [file 12978_2025_1978_MOESM1_ESM.docx]

**ANNEX DOCUMENT 1. NFHS-5 contraceptive method descriptions**

FEMALE STERILIZATION: A woman can have an operation to avoid having any more children.

MALE STERILIZATION: A man can have an operation to avoid having any more children.

IUD OR PPIUD: A woman can have a loop or coil placed inside her vagina by a doctor or a nurse.

INJECTABLES: A woman can have an injection by a health provider that stops her from becoming pregnant for one or more months.

PILL: A woman can take a pill every day or every week to avoid becoming pregnant.

CONDOM OR NIRODH: A man can put a rubber sheath on his penis before sexual intercourse.

FEMALE CONDOM: A woman can place a sheath in her vagina before sexual intercourse.

EMERGENCY CONTRACEPTION: A woman can take pills up to three days after sexual intercourse to avoid becoming pregnant.

DIAPHRAGM: A woman can place a diaphragm inside herself before intercourse.

FOAM/JELLY: A woman can place foam or jelly inside herself before intercourse.

LACTATIONAL AMENORRHOEA METHOD (LAM): Up to six months after childbirth, before the menstrual period has returned, a woman uses a method requiring frequent breastfeeding day and night.

RHYTHM METHOD: Every month that a woman is sexually active she can avoid pregnancy by not having sexual intercourse on the days of the month she is most likely to get pregnant.

WITHDRAWAL: A man can be careful and pull out before climax.
